# An integrated photonics engine for unsupervised correlation detection

**DOI:** 10.1126/sciadv.abn3243

**Published:** 2022-06-01

**Authors:** Syed Ghazi Sarwat, Frank Brückerhoff-Plückelmann, Santiago García-Cuevas Carrillo, Emanuele Gemo, Johannes Feldmann, Harish Bhaskaran, C. David Wright, Wolfram H. P. Pernice, Abu Sebastian

**Affiliations:** 1 IBM Research Europe, Säumerstrasse 4, 8803 Rüschlikon, Switzerland.; 2 Center for Soft Nanoscience, University of Münster, Busso-Peuss-Str. 10, 48149 Münster, Germany.; 3 Department of Engineering of Engineering, University of Exeter, Exeter EX4 4QF, UK.; 4 Department of Materials, University of Oxford, Oxford OX26HT, UK.

## Abstract

With more and more aspects of modern life and scientific tools becoming digitized, the amount of data being generated is growing exponentially. Fast and efficient statistical processing, such as identifying correlations in big datasets, is therefore becoming increasingly important, and this, on account of the various compute bottlenecks in modern digital machines, has necessitated new computational paradigms. Here, we demonstrate one such novel paradigm, via the development of an integrated phase-change photonics engine. The computational memory engine exploits the accumulative property of Ge_2_Sb_2_Te_5_ phase-change cells and wavelength division multiplexing property of optics in delivering fully parallelized and colocated temporal correlation detection computations. We investigate this property and present an experimental demonstration of identifying real-time correlations in data streams on the social media platform Twitter and high-traffic computing nodes in data centers. Our results demonstrate the use case of high-speed integrated photonics in accelerating statistical analysis methods.

## Teaser

Integrated photonic computational memory engine performs difficult statistical method computations in real time.

## INTRODUCTION

The use of statistical methods to detect patterns and model structures in data streams is becoming increasingly pervasive in today’s era of artificial intelligence and the Internet of Things (*1*–*4*). To that end, various dedicated algorithms and computing hardware using application-specific integrated circuits and graphics processing units have emerged (*5*–*7*). Nonetheless, these approaches continue to operate on the memory-wall bottlenecked digital von Neumann architecture, where data need repeated shuttling on power-hungry interconnects. This presents a limit on the achievable computational speed and incurs notable energy costs. More specific to data analytics, the need to constantly shuttle information also implies that data’s real-time or temporal correlation aspects can be lost. Determining the temporal correlation in and between data streams is important for a host of applications, ranging from social media analysis to financial forecasting, the detection of hacking threats, and much more. One approach for minimizing data movements within computer systems, so facilitating correlation detection, is the scheme of computational memory, where certain computations can be performed in the same physical location as where the data are stored. This can be realized using the physical attributes of memory devices (*8*, *9*), such as nonvolatile memories, including phase-change memories. However, because even these approaches use conventional electronic addressing and components, they still remain limited in bandwidth and energy and lack the intrinsic potential for parallelizing operations (except by physical replication of hardware). An intrinsic parallelization capability is particularly relevant and required for data-intensive workloads.

Integrated photonics offers improvements in computational memory by enabling parallel data transfers through wavelength division multiplexing (WDM) and by providing extremely high data modulation speeds. More recently, these characteristics have been leveraged in photonic tensor cores for accelerating deep learning. In these approaches, phase-change materials are used as attenuating matrix elements (*10*–*15*) that absorb a desired amount of light depending on their amorphous-crystalline fraction (phase configuration). The property of phase-change materials that is used is the ability to program them to multitransmissive states, which is achieved through partial amorphization pulses (*16*, *17*). Here, we demonstrate a “photonics computational memory” engine for big data analytics, where we leverage yet another property of phase-change materials—that is, the accumulative behavior arising from the crystallization dynamics (*18*, *19*). We investigate this property through an experimental theory framework and demonstrate photonics computational memory for the demanding task of unsupervised correlation detection. Specifically, we demonstrate two use cases: The first is correlation detection on social media, through examples of Twitter and sentiment analysis, and the second is of anomaly detection in high-traffic data centers.

## RESULTS

### Crystallization dynamics and photonic computational memory

**Fig. 1.**
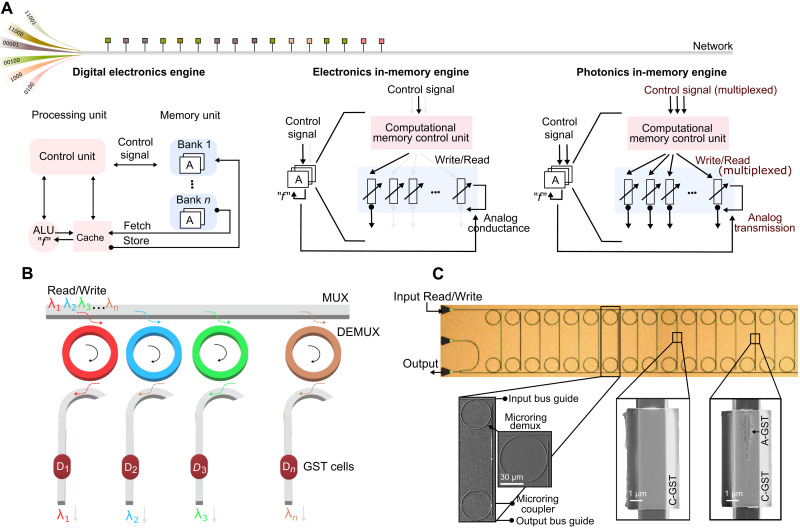
Concept of an integrated photonic computational memory. (**A**) A comparison of a digital electronic engine, computational memory electronic engine, and our computational memory photonics engine, for processing data streams for a correlation detection operation. Digital electronics require many sequential processing steps, in which data are shuttled back and forth between the memory and the processing unit that comprises an arithmetic logic unit (ALU). In electronic computational memory, suitable electrical signals are sequentially applied to the memory devices in correspondence to correlations between data streams. The conductance of the devices evolves in accordance with the electrical input, and the result is sequentially retrieved by reading individual devices. Photonics computational memory has wavelength multiplexing as an additional degree of freedom, enabling parallel write and read operations on multiple devices. Here, the transmission of the devices evolves in accordance with the optical input. (**B**) A conceptual illustration of an integrated photonic computational memory engine to compute correlations. (**C**) The top panel is an optical micrograph of a fabricated device array. The bottom panel shows a zoomed-in view of the various building blocks, including the phase-change memory in two structural states of GST phase-change material (C-GST and A-GST represent the crystalline and amorphous states of GST, respectively).

In a departure from electronic accelerators, our photonic computational memory engine performs many parallel correlation detection operations using multiple wavelengths (see Fig. 1A). The computational elements in our photonics engine are photonic memory devices using 10-nm thin film of optically tunable Ge_2_Sb_2_Te_5_ (GST) phase-change material, capped by 10-nm SiO_2_. The devices can be toggled between the transmissive-amorphous and absorptive-crystalline states of GST (see sections S1 to S3). This is achieved by near-field coupling of the electromagnetic waveguide mode to either SET (crystallize) or RESET (amorphize by melting over ∼600°C and quenching) the phase-change material.

Another characteristic property of phase-change material is that these structural changes can be progressively achieved (accumulated) using the material’s crystallization (or amorphization) dynamics (*20*–*24*). In this accumulation scheme, the device’s transmission evolves in accordance with the number of (constant amplitude) crystallization pulses that encode a computational problem. Moreover, the result of the computation gets stored in place, a property that we exploit in this work.

Our photonics circuit comprises three blocks: a wavelength multiplexer (MUX), which is an optical waveguide for inputs; demultiplexers (DEMUX), which are carefully tuned ring resonators; and waveguides with integrated GST cells (see Fig. 1B). Experimentally, such a circuit can be realized through different approaches. One is shown in the micrographs of Fig. 1C, where we use a double-ring configuration with connection waveguides. Such a design delineates the effect of phase transitions in the GST cells from the resonance wavelengths (the optical coupling between MUX/DEMUX and resonators). Here, a photonics core comprises multiple GST cells, and many such cores are distributed on the measurement chip. Each GST cell, through its DEMUX, can drop a selected wavelength-multiplexed signal from the MUX. Thus, many devices can be addressed with minimal cross-talk (<−10 dB) and in parallel for both READ and programming operations (see section S3). For clarity, in what follows, we refer to write as SET and to erase as RESET (note that the literature uses a reverse nomenclature for the write and erase operations).

**Fig. 2.**
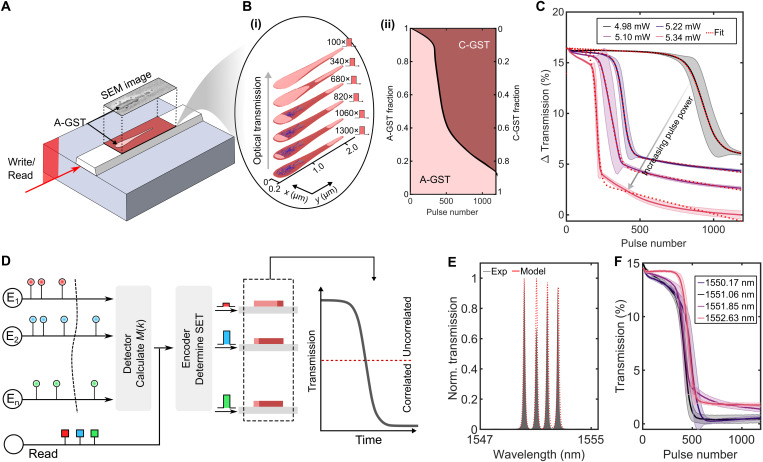
Crystallization dynamics in GST cell for correlation detection. (**A**) An illustration of a photonic GST cell. The crystalline(C)-GST patch is represented in red, with a streak of amorphous mark that is shaped as is noted in the experiments. SEM, scanning electron microscopy. (**B**) (i) Finite element and phase-change modeling of the accumulative crystallization process of the amorphous GST region. The plot illustrates the different phase configurations produced by a train of identical 200-ns-wide 4.1-mW crystallization pulses. With more and more crystallization pulses, the crystalline fraction grows, as well as new crystalline grains nucleate within the amorphous volume (colored blue). (ii) Accumulative crystallization behavior of a GST cell based on the simulated results shown in (i). (**C**) Experimental accumulative crystallization behavior of a GST cell. The different traces represent distinct experiments, each with a different crystallization pulse power. The optical transmission progressively decreases from crystallization, and the number of accumulations increases for lower pulse powers. The red dotted lines are fit the data. (**D**) In our photonics engine, each event is assigned to a GST cell and with a unique input wavelength for read and write operations. Whenever a spike is detected in an event’s data stream, write pulses are applied to the allocated devices. The sum of all events at any instance is used to modulate the width of the write pulses. During the analysis, the transmission in the devices change, which are used to detect correlations. (**E**) Transmission spectra of four GST cells used for multiplexing. Each one has a unique resonance wavelength that overlaps minimally with the others. The red dotted lines represent the spectra estimated by our circuit model. (**F**) Accumulative behavior in four GST cells, implemented using the WDM property with pulses encoded in the four wavelengths shown in (E).

When a SET pulse is applied to a GST cell that has an amorphous volume *u*_v_, a part of the amorphous region (Δ*u*_v_) crystallizes at a rate mainly dictated by the temperature-dependent crystal growth velocity of GST. It is thus the movement of the crystalline/amorphous interface that determines the changes in crystallinity in the device. To understand these characteristics, it becomes important to account for the interplay of three physical processes in the device (*25*), namely, optical, thermal, and phase-change. Noting this and using finite element and analytical methods, we construct a theoretical framework for the photonic GST cells. For any phase configuration of GST, the framework estimates the temporal temperature profile in the cell produced by a SET pulse, and via this profile, the position and evolution of the amorphous/crystalline interface (see Section S4). In Fig. 2A, we present an exemplar case where the crystallization dynamics of the amorphous mark noted in our SEM micrograph (see Fig. 1C) are investigated. Each SET pulse is observed to induce crystal growth nucleation, and this is noted to occur because of thermal heating of amorphous volume, via optical absorption by the more absorptive surrounding crystalline volume. Figure 2B (i) illustrates the phase configuration of the cell under increasing levels of accumulations. Specifically, for every SET pulse, we observe crystallization to proceed inward from amorphous/crystalline interfaces, where optical heating is maximum, and the process to repeat nonlinearly for increasing number of SET pulses [see Fig. 2B (ii) and section S3]. These results suggest, in line with a general understanding of nucleation-growth materials science, that crystallization occurs with an initial stage of growth that is approximately exponential; then, as it saturates, the growth slows. Thus, the crystallization process, as a function of number of SET pulses (*k*), can be modeled using a logistic (sigmoidal) expression of the form $\frac{{\mathrm{du}}_{v}}{\mathrm{dk}}\propto \kappa u_{v}(1-\frac{u_{v}}{u_{v^{\prime }}})$ to describe Δ*u*_v_. Here, κ is the rate, governed by the power in the SET pulse, and *u*_v^′^_ is the maximum allowable crystal volume.

We experimentally investigate this behavior by performing optical accumulations in a 4-μm-long GST cell. The measurement is performed by programming an amorphous volume in the cell using a single 8-mW RESET optical pulse of 200-ns duration and then progressively crystallizing the amorphous mark using 200-ns-wide identical SET pulses. We repeat the measurement for SET pulses of increasing optical power (see Fig. 2B). In our measurements, the optical transmission (*T*_0_) of the cells in the fully amorphous phase configuration is defined as the baseline of the transmission readout. Any subsequent change of the readout (Δ*T* = *T* − *T*_0_) during the measurement is normalized as the relative change in percentage ($\frac{\Delta T}{T_{0}}$) to the baseline (each data point in this plot is an average of 50 measurements). The results show that optical transmission in the cell decreases with the increasing number and power of SET pulses. This occurs because under either case, more and more absorptive-crystalline volume forms at the expense of the transmissive-amorphous volume. We do observe that the crystallization follows a sigmoidal behavior ($\frac{{\mathrm{du}}_{v}}{\mathrm{dk}}\propto \frac{d\Delta T}{\mathrm{dk}}$) and that it is tunable using pulse power as a control variable (the dotted red traces represent fit to the experimental data).

### Correlation detection

In a generic formulation, the problem of correlation detection requires the computation of means, variances, and covariances in a format $c=\frac{\text{Cov}[X_{i}(k),X_{j}(k)]}{\sqrt{\text{Var}[X_{i}(k)]\times \text{Var}[X_{j}(k)}]}$, where *X_i_* and *X_j_* are discrete binary (1 and 0) stochastic events between which correlations (*c*, with *c* = −1 to 1) at time instance *k* are computed. Cov is the covariance [$\text{Cov}=\frac{(X_{i}(k)-\bar{X_{i}})\times (X_{j}(k)-\bar{X_{j})}}{n-1}$, where $\bar{X_{i}}$ is the mean of the *i*_th_ input data stream, and *n* is data size]. In a matrix-based approach on a von Neumann machine, for *N* distinct events, correlations are computed *N*^2^ times, either iteratively or recursively. In a computational memory module, we simplify the task by using the crystallization dynamics of the GST cells. In this scheme, we map the correlation coefficients to the transmission states of the devices (*c* ↦ *T*), such that devices with similar transmissions states are grouped and correlated under the condition that the transmissions are below an arbitrarily set threshold.

At each time instance *k*, a collective momentum [*M*(k)] is estimated, which gathers the instantaneous sum of all *N* distinct events [$M(k)=\sum_{j=1}^{N}X_{j}(k)$]. This operation essentially counts the number of 1’s in the binary stream. However, because this operation is based on temporal coding, if the inputs are rate mismatched, *M*(*k*) can be wrongly dominated by the events of high input rates. Therefore, the input event streams are subjected to averaging for rate normalization, i.e., $X_{j}(k)=\{\begin{array}{ll} 1 & \text{if}\;X_{j}(k)⊙\text{exp}\;(-\alpha \times k)>0.5\\ 0 & \text{Otherwise} \end{array},\text{where}\;j=1\;\text{to}\;N,\text{and}\;\alpha$ is a number between 0 and 1. Following the *M*(*k*) estimation, identical write pulses are applied to all GST cells, for which the binary event has a value of 1. Here, *M*(*k*) is linearly mapped to the number of write pulses [*N*_SET_(*k*) = γ × *M*(*k*), where *N*_SET_ is the number of write pulses and γ is a positive number]; however, mapping can also be done to the amplitude of the pulse [*A*_SET_(k) = γ × *M*(*k*), where *A*_SET_ is the amplitude of the optical pulse (in milliwatts)]. For every unique *M*(*k*), the corresponding devices crystallize to an extent dictated by the crystallization dynamics. It can be shown that after *K* time steps, Δ*u*_v_ in the *i*_th_ GST cell will be $\Delta u_{v_{i}}(K)=\sum_{k=1}^{K}\delta u_{v_{i}}(k)X_{i}(k)$, where $\delta u_{v_{i}}(k)=N_{\text{SET}}(k)\times v_{g}(T)=\gamma v_{g}(T)\sum_{j=1}^{N}X_{j}(k)$. For the correlation between event pairs, $\Delta u_{v_{i}}(K)=\gamma v_{g}(T)\sum_{j=1}^{N}\sum_{k=1}^{K}X_{i}(k)X_{j}(k)$. Thus, if *X_i_* belongs to a correlated group, Δ*u*_v*_i_*_(*K*) will be large, yielding smaller optical transmission and vice versa. By simply using the transmission as a proxy classification metric, correlated GST cells can be clustered from the uncorrelated ones (*20*). Note that there is similarity of this approach with the generic formulation described earlier. Δ*u*_v*_i_*_(*K*) can be rewritten to the form $\Delta u_{v_{i}}(K)=K\gamma v_{g}(T){\hat{W}}_{i}$, where ${\hat{W}}_{i}$ is an estimator of correlations. It is a numerical weight calculated by summing the elements along the row or column of the uncentered covariance matrix. If a process *X_i_* belongs to a correlated group, then $E[{\hat{W}}_{i}]=(N-1)p^{2}+p+(N_{c}-1)\mathrm{cp}(1-p)$, where 0 ≤ *p* ≤ 0.5, *c* is the correlation coefficient, and *N_c_* is the number of correlated processes. It can be also shown that $\text{Var}[{\hat{W}}_{i}]=E[{\hat{W}}_{i}^{2}]-E{\left[{\hat{W}}_{i}\right]}^{2}\leq \frac{N^{2}}{4K}$. Thus, by monitoring the estimator in the limit of large *K*, correlated processes can be determined, and with increasing values of *c*, it becomes easier to determine these processes. This is equivalent to our in-memory approach, where the integral of *M*(*k*) and, thus, Δ*u*_v*_i_*_ are the estimators of correlations.

In our correlation engine (see Fig. 2D), each event is assigned to a GST cell, and a distinct wavelength (λ*_n_*) for parallel computing. We experimentally developed such a framework. Figure 2E illustrates normalized frequency spectra of a correlator with four 4-μm-long GST cells. By design of DEMUX units, each GST cell is allowed to pick only a specific wavelength, independent of the phase configuration of GST (see section S3). Thus, in the investigated range, there are four minimally overlapping resonance wavelengths, each distinct to a unique GST cell. This, thus, enables parallel multiplexing for read and write operations. Figure 2F further illustrates this. The plot shows simultaneous accumulation measurements performed, via WDM, on the four cells (each data point is an average of 50 measurements). In these measurements, the cells are first amorphized using a single 200-ns-wide 8-mW RESET pulse and then progressively SET using identical 200-ns-wide 5.10-mW pulses, using the highlighted wavelengths. It is clear from these figures that our devices offer the capability to fully operate in parallel for independent or dependent programming and read operations. It is also noteworthy to emphasize that the crystallization dynamics are such that they provide an intrinsic computational nonlinearity (logistic type) in each cell: The devices have the ability to classify decisions in place based on their transmission values, without requiring digital activation units. In an attempt to simulate a prototype phase-change photonic computational memory engine based on the device characteristics discussed so far, we built a correlator model simulating our chip design and devices within the framework of a commercial photonic integrated circuit simulator IPKISS (see section S4 and data fit colored as red in Fig. 2E).

### Anomaly and intrusion detection in computer networks

As a first example, using our photonics correlation engine, we demonstrate the identification of events that deviate from some standard (logged) behavior—a computational problem referred to as anomaly detection or outlier analysis (*26*, *27*). Anomaly detection becomes particularly relevant (and challenging) in high-traffic data transfer settings, such as across processing nodes in hybrid (or multicore) computing machines and data servers. Generally, the goal of analysis is to indicate critical incidents, such as software glitches, hardware malfunctions, or potential “hostile” intrusions.

**Fig. 3.**
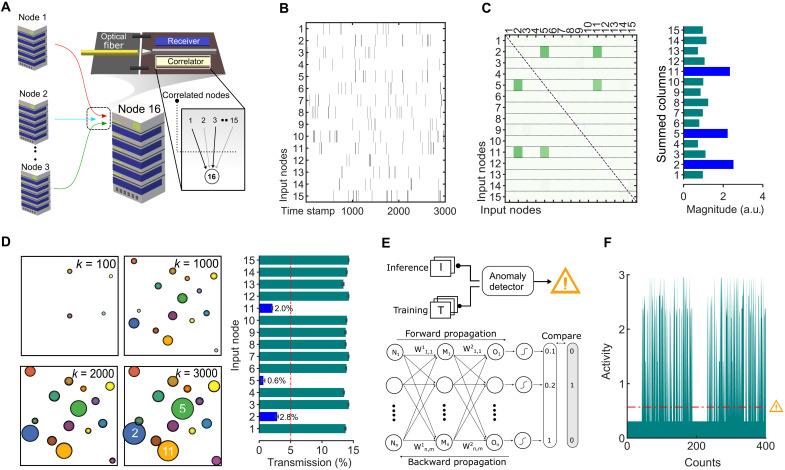
Anomaly detection in high-traffic data nodes using photonic computational memory. (**A**) A conceptual illustration of high-speed data communication between computing nodes, such as servers in data centers. Each node is equipped with a transceiver unit that houses a correlator unit based on GST cells. The inset shows that at any time, certain nodes are correlated with respect to a reference node. (**B**) A raster plot illustrating event-based data streams communicated from various nodes to node 16. (**C**) The left panel is a standard covariance matrix of the data streams. The right panel, which is computed from the matrix, shows that certain nodes are correlated. a.u., arbitrary units. (**D**) The left panel shows four count plots computed by the photonics computational memory module at increasing time instances. The right panel illustrates the transmission states of the GST cells associated with each node. By monitoring the transmissions, the correlated nodes are estimated. (**E**) The use of the photonics engine as an anomaly detector. This is realized by comparing correlations to a logged pattern learned using a two-layer artificial neural network. (**F**) Illustration of anomaly detection. A spike in a time series signal is indicative of an abnormality and registered as an anomaly if it is larger than a set threshold (red dotted line).

In Fig. 3A, we sketch a schematic of a data center that houses multiple interconnected data servers (nodes). The data servers communicate with each other and the web using optical cables and integrated photonics transceiver. To such a transceiver unit, we add the correlation detection (correlator), whose role is to perform data aggregation and analysis. At any time instance, the correlator logs the correlations between the input data streams, for example, from *N* − 1 nodes to the *N*^th^ node. For the purpose of demonstration, we created artificial datasets containing correlated and uncorrelated input data streams using Poisson statistics. If *X_i_*(*k*) is an independent random variable in an event, it has probabilities of *P*[*X_i_*(k) = 1] = *p*, and *P*[*X_i_*(*k*) = 0] = 1 − *p*, and is correlated to other events with the expression (*20*, *28*) *p*^2^ + *c*_*i*, *j*_ × *p*(1 − *p*) − *p*^2^. Figure 3B is a raster plot highlighting the event-based data stream input to the node 16 (arbitrarily chosen) in our data center illustration. All data streams have the same rate; however, only some are correlated. This is to say that their 1’s (black vertical lines) and 0’s (white background) appear at node 16 at identical time instances. In Fig. 3C, by iteratively using the standard algorithm 1 on a standard electronic computer, we plot an uncentered covariance matrix to illustrate the different positively correlated nodes in the investigated time window. We project the correlation coefficients described in this matrix onto a two-dimensional plot by simply summing the matrix elements along the columns (see Fig. 3C, right), where each column is distinctive for an input node (1 to 15). The magnitudes for nodes 2, 5, and 11 are similar and the largest, suggesting that they are correlated, when communicating with node 16.

Our goal is to now reproduce this result by exploiting the accumulative property of GST in the photonics correlation engine. In our measurements, we assign each computing node to a distinct GST cell, and all cells are initialized to an amorphous phase configuration using a single 200-ns-wide 8-mW RESET pulse. Because of our experimental limitations to incorporate more than four simultaneous wavelengths, we exploit parallel accumulations in four GST cells. By sequentially repeating then the experiment four times, we gather data on accumulations (using 200-ns-wide 5.10-mW SET pulse) in 16 devices, such that each computing node is represented with a unique accumulation trace. Figure 3D illustrates a count plot that is computed by the computational memory algorithm for increasing time steps (*k*). Each colored circle represents a GST cell, and the size encodes correspondingly the number of accumulations that the cell has experienced. Note that accumulative SET pulses are only applied when at least two GST cells are correlated (γ = 7). Thus, as a function of *k*, correlated devices appear as larger circles. For *k* = 3000, we have plotted the optical transmissions in all the GST cells on the right panel. The devices corresponding to nodes 2, 5, and 11 have the lowest transmissions, i.e., most number of accumulations. On the basis of the chosen threshold value of 5% transmission (red dotted line), we can thus classify these nodes to belong to a correlated group, as was predicted by the conventional uncentered covariance matrix approach on electronic hardware. Note that at no point during this computation was any data shuttled or processed. Using the attribute of accumulative crystallization, the devices’ transmission state smartly converged toward to the right solution.

It is noted that the correlated and uncorrelated nodes may well change over time or with the workload. This may occur in ways that are predictable. To illustrate this, we train a standard two-layer artificial neural network with synthetic data streams to learn the communication pattern between the 16 nodes (see Fig. 3E). During operation (inference), the photonics engine is set up to experimentally classify correlated and uncorrelated nodes in the above described format as well to compare (denoted as activity) the computations against an expected pattern. The engine thus serves as an anomaly detector, and at any time that the computed pattern differs markedly, it indicates an anomaly, because the activity spikes. This is illustrated in Fig. 3F: Each spike is a deviation from the logged behavior, and if it takes a value greater than the set threshold (red dotted line), then it is classified as an anomaly.

### Social media analysis: Sentiment detection

The second example that we discuss is for social media analytics. In social media, the goal of correlation detection is often to transform unstructured data into useful information. This is important in a range of tasks, including financial market, sentiment (and opinion), and security analyses. The task chosen here is to find the correlations between specific words in a collection of tweets posted on the platform Twitter (*29*), such that a consensus (and opinion) on a subject can be reached.

**Fig. 4.**
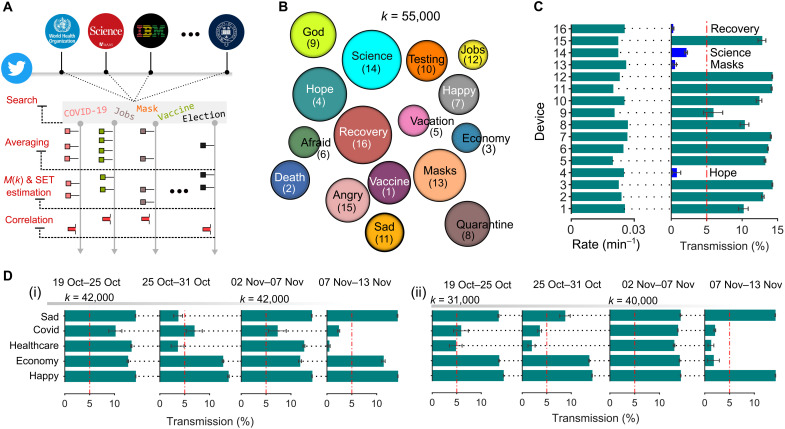
Social media and sentiment analysis using photonic computational memory. (**A**) An illustration of correlation detection on the social media platform Twitter. A keyword-based event stream is generated for every post on Twitter, and the temporal correlations between all posts are detected by the photonics computational memory engine. (**B**) A count plot computed by the photonics computational memory module for 55,000 tweets on the subject of COVID-19. (**C**) The left panel is a histogram illustrating the rate of binary streams at the detector, and the right panel shows the transmission states of the GST cells. The devices (thus keywords) with low transmissions are correlated. (**D**) Sentiment analysis for the 2020 U.S. election candidates using tweets. Some GST cells are allocated to words describing generic human emotions, and their transmission states are used as a measurement of sentiments.

We demonstrate just that using our photonics correlation engine. Figure 4A illustrates the scheme that we use for finding correlations between tweets. Using Twitter’s application programing interface developer platform, our computational memory grabs live tweets and processes each tweet with a local search algorithm for locating selected keywords. We take a bag-of-words approach (see section S5) to locate the keywords in tweets, and every keyword has many equivalent meaning proxy words. Each keyword is regarded as an independent binary event (1’s and 0’s) that produces a data stream. The data streams, however, can have significant rate variations because some words are more commonly used than others. When this occurs, the high-rate channels can obstruct the learning of pairwise correlations. We avoid this by using a temporal filter, which normalizes the rate across all channels through averaging (α = 0.01). Both high- and low-rate channels thus contribute equally to correlations, and *M*(*k*) that encodes SET pulses is more faithfully estimated. We now show correlation detection between tweets posted in late 2020 on the subject COVID-19. There are 16 keywords of interest, and each keyword is associated with a unique GST cell (labeled as numbers). As previously, because of the experimental limitations to incorporate more than four simultaneous wavelengths, we exploit parallel accumulations in four GST cells and repeat the measurement four times to gather the accumulated states of 16 devices. All devices are initialized to an amorphous state, and a crystalline volume is progressively accumulated using a 200-ns-wide 5.10-mW SET pulse (γ = 10). Figure 4B illustrates a count plot for 55,000 tweets (*k* = 55,000). Each circle represents a GST cell and encodes, via its size, the number of accumulations. When the measurement is complete, four GST cells, namely, 4, 13, 14, and 16, converge into a group of devices with the most accumulations. The left panel of Fig. 4C illustrates the rate histogram of the input data streams that produce this result. All events have similar input rates, and thus, the correlations must only arise if the events are temporally related. The transmission states of all the cells corresponding to the count plot are shown in the right panel of Fig. 4C. Notably, the optical transmission through the cells with the most accumulations is the smallest and below the chosen threshold value (red dotted line). These devices (keywords) are therefore related by temporal correlations. The correlated group comprises keywords of “hope,” “masks,” “science,” and “recovery,” which broadly match the online engagements in late 2020 on the vaccine discovery and COVID-19 preventive measures.

In addition, if every keyword is associated with a sentiment, either positive, negative, or neutral, then correlation detection can be used for scoring sentiments (sentimental analysis). We illustrate this with an example of Twitter analysis during the U.S. 2020 election campaigns. In Fig. 4 (D and E), we plot the transmission states of five GST cells for four different time periods. The measurements are performed in the format described above (α = 0.015 and γ = 15); however, because we limit to five devices, we repeat the measurement with four multiplexed devices twice. For the Republican candidate, the transmission maps are shown in Fig. 4D (i), where each analysis shows the results of 42,000 tweets. For the Democrat candidate, the transmission maps during the same time periods and with a comparable number of tweets are also shown in Fig. 4D (ii). The cells are ascribed to important keywords (subjects) that the candidates were associated with during their campaign. The cells associated with the words sad and happy are used for scoring negative and positive emotions, respectively. These sentiments are registered only if the transmission states of the corresponding devices are below the set threshold (red dotted line) at the end of the experiment.

## DISCUSSION

We have shown that the use of integrated photonic computational memory can provide a powerful computational platform for the direct hardware solution of statistical problems in the optical domain. Compared with conventional electronic approaches, our phase-change all-optical engine benefits from the parallel multiplexing capability and the high speed and high bandwidth inherent to optical systems. Specific to the correlation detection problem, for *N* data streams, the computational time complexity is reduced to *O*(1) from the *O*(*N*^2^) covariance-based CMOS approach, thus making it possible to handle large amounts of data in a short amount of time. This is also an improvement compared to electronic computational memory that operates with *O*(*N*) time complexity. The linear scaling with the number of input originates from sequential electronic addressing (write and read) because of peripheral circuity and interconnect resistive heating limitations. Additional challenges in the electronic domain arise in the implementation because of issues with limited number of programmable nonvolatile states, device variability, cyclability, and drift (*21*, *30*). Besides improving on these issues (*16*, *17*), the photonic WDM approach allows parallelized data addressing, such that the complexity reduces to *O*(1) for any input size. Moreover, the photonic computational memory approach benefits from high bandwidths, limited only by the crystallization speed and thermal time constant of GST cell. Furthermore, the use of WDM can be further extended so that a given set of wavelengths can be used across multiple tiles of GST cells in a layered circuit architecture (see section S6). Each tile can be associated with the same process (application) or with different processes. The latter would imply that correlation in *M* number of processes, each with *N* data streams, can be also found with *O*(1) time complexity. We wish to emphasize that our computational memory approach provides a more generic solution to temporal correlation detection, that is, it is limited to the case where the goal is to determine (or cluster) correlated *c* > 0 processes having a binary-type data stream. The approach thus maps the increasing values of *c* to a larger separation between the correlated and uncorrelated devices, and vice versa (see section S5). The negative correlation coefficients get disregarded in our estimation of *M*(*k*), which averages over all processes, as opposed to working on a per-event (device) level.

Some important challenges should be pointed out when considering scaling up our photonics approach. The first is fabrication imperfections and optical losses. Spectral overlaps from variations in the radii of microring resonators and dissimilarities in the coupling ratios due to fluctuations in resonator-bus waveguide gaps are an example of the former. The latter would include propagation (scattering) and absorption in the waveguides and phase-change materials, and coupling losses at the MUXs and DEMUXs. Both imperfections and losses are expected to increase with the number of devices used in the correlator (see section S6). The second challenge is the device’s areal footprint. Each photonics device in this work is ∼1000 μm^2^ in size, which is large compared to <∼1 μm^2^ electronic counterparts. However, this difference can be compensated to some extent by the higher accumulate density (number of accumulations per device, which are ∼40× times more than in electronic cells) and by scaling down the photonic circuitry using higher refractive index materials and an optimized design layout. For example, by using Si instead of SiN, the device footprint can be reduced by ∼100×. Nonetheless, we note that among others, some high-bandwidth applications, such as computing nodes in data centers, very long baseline telescopes, and particle accelerators, which require a modest number of devices, can still be accelerated with our approach (see section S6 discussing 256 devices).

In summary, we have demonstrated the first instance of a photonic computational memory platform that learns data in real time using statistical methods. The photonic engine jointly exploits the accumulative property in GST cells and WDM of optics for colocated data processing and storage. We investigated these properties using experimental and theoretical methods. We built experimental chips and developed finite elemental and analytical frameworks for modeling accumulation in photonic memories, which we then exploited for system-level simulations. Crucially, the transmission of the nonvolatile GST cells, receiving correlated inputs, evolves to a low value, and by monitoring these transmission values, we detect temporal correlations using a computational memory algorithm. To illustrate the use case of this approach, we presented experimental demonstrations of identifying real-time correlations in data streams on the social media platform Twitter for social media analysis and for threat and anomaly detections in high-traffic computing nodes in data centers. Our results set another example for the potential of integrated photonics for performing challenging computational problems more efficiently.

## MATERIALS AND METHODS

### Fabrication

The photonic circuits were fabricated using electron beam (e-beam) lithography with a 100-kV system (Raith EBPG 5150). The process flow is as follows. (i) Opening windows for liftoff using positive tone resist polymethylmethacrylate (PMMA) on a silicon wafer (Rogue Valley Microdevices) with 3300-nm silicon oxide and 330-nm silicon nitride layer on top. Development using 1:3 methyl isobutyl ketone (MIBK):isopropanol for 2 min, and deposition of a stack of 5-nm chromium and 120-nm thin gold using e-beam physical vapor deposition for e-beam marker. Liftoff step to remove the PMMA using acetone. (ii) A second lithography step using a negative-tone e-beam resist arN 7520.12 to pattern photonic structures. We used prebaking conditions of 85°C for 60 s, followed by development in MF-319 solution for 75 s, following which a postbake treatment at 85°C for 60 s. Using reactive ion etching in a CHF_3_/O_2_ plasma chemistry, the resist mask is transferred onto the sample. A posttreatment in oxygen plasma for 10 min to remove any remaining resist. (iii) A final e-beam lithography step for patterning the windows for the deposition of the phase-change material (PCM). This is performed using a positive resist and follows the same step as in (i). After development, 10 nm of the PCM GST is sputter deposited and covered by a 10-nm thin film of SiO_2_ [5-mtorr working pressure, 15–standard cubic centimeters per minute (sccm) Ar, 30-W radio-frequency power, and 2 × 10^−6^ torr base pressure] to prevent oxidation of the GST. Prior to the experiments, the chip is treated on a hot plate at 250°C for approximately 15 min to crystallize GST.

### Measurement setup

The setup is operated at telecommunication wavelengths (C-band) using tunable laser sources. The individual laser sources for pump and probe pulses are spectrally aligned to the resonance wavelengths of the ring resonator–based on-chip multiplexer and applied to the chip using single-mode optical fibers with ferrule core/angled physical contact connectors (supplier: Thorlabs). Briefly, the probe lasers (Santec, TSL 510) are guided in (from the left in the figure)/out (to the right in the figure) of the optical chip by two circulators (OC) using polarization control blocks. Coupling to the chip is done in free space using angled fiber arrays and on-chip Bragg diffraction gratings. At the output, the read pulses are detected by a slow 100-kHz photodetector (s-PD; supplier: New Focus Model 2011) that is connected to a digital-to-analog converter and computer. The pump-pulsed lasers generated using a 500-MHz arbitrary function generator (arbitrary waveform generator; supplier: Agilent, HP 8131A) follow the opposite trajectory (from the right to left in the figure). The pulses are polarization controlled, temporally modulated using a 10-GHz electro-optic modulator (EOM; supplier: Lucent 2623), amplified by erbium-doped fiber amplifier (EDFA; supplier: Pritel LNHPFA-33), and spectrally filtered (optical transfer function filter; supplier: Pritel TFA-1550) before getting injected into the chip using an OC. At the output, the pulses are detected by a fast photodetector (f-PD; supplier: New Focus Model 1811) after passing through a variable optical attenuator (VOA) that safeguards the detector. The pulses are visualized on a 1-GHz oscilloscope. For single-device measurements, we used pump pulses to the wavelength 1560 nm and write pulses to the wavelength 1550 nm. All measurements were carried out in standard room ambient conditions.

## Supplementary Materials

### This PDF file includes:

Sections S1 to S7 Figs. S1 to S7
