# Supplementary material for: An integrated photonics engine for unsupervised correlation detection
Source: Sci Adv. 2022 Jun 1;8(22):eabn3243. doi: 10.1126/sciadv.abn3243 (PMC13155488; doi:10.1126/sciadv.abn3243)
Supplement: Supplementary file 1 — Sections S1 to S7 Figs. S1 to S7 [file sciadv.abn3243_sm.pdf]

Science Advances
AAAS

Supplementary Materials for

An integrated photonics engine for unsupervised correlation detection

Syed Ghazi Sarwat *et al.*

Corresponding author: Syed Ghazi Sarwat, ghs@zurich.ibm.com; Abu Sebastian, ase@zurich.ibm.com

Sci. Adv. **8**, eabn3243 (2022)
DOI: 10.1126/sciadv.abn3243

The PDF file includes:

Sections S1 to S7
Figs. S1 to S7

Other Supplementary Material for this manuscript includes the following:

Movie S1

Section S1. Device fabrication

The steps are detailed in the Methods section of the Manuscript.

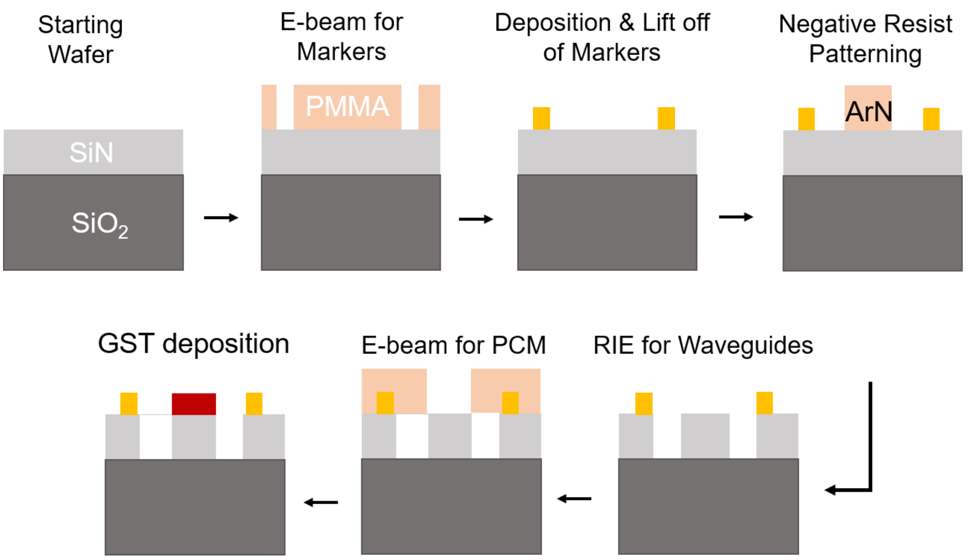

Figure S1: Fabrication scheme. Process flow of our photonic circuit fabrication with standard lithography methods.

Section S2. Measurement setup

The steps are detailed in the Methods section of the Manuscript.

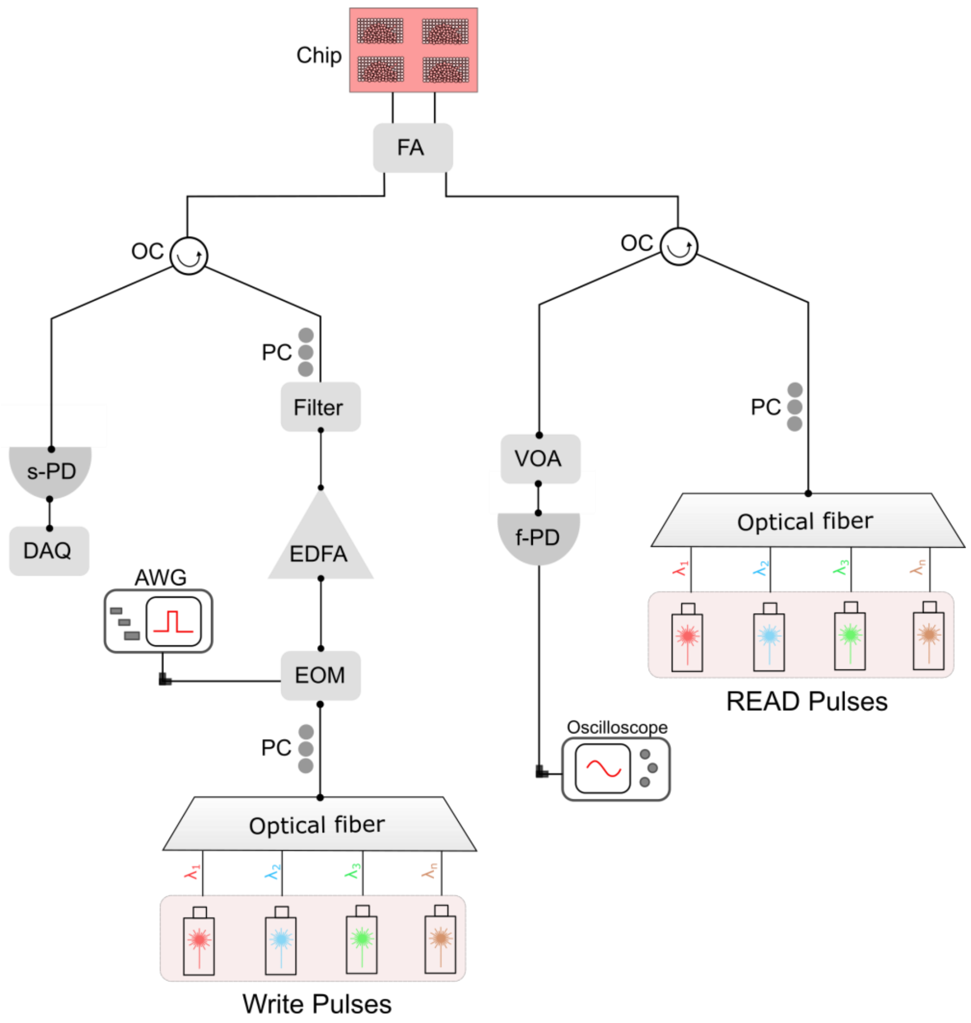

Figure S2: Optical measurement setup. An illustration of the Read/Write measurements scheme.

Section S3. Device Components and losses

Ring Resonators

A schematic of our photonic device structure is shown in Figure S3a. For multiplexing the important control parameters are the gap sizes between the input -waveguide and -resonator, and the output (drop) -resonator and waveguide (shown by red arrow) and the ring resonators' radii (shown by black arrow). For the former, it is can be shown that maximal transmission can be realized when input gap=output gap or the device has a symmetric configuration. Our devices are therefore symmetric. For maximal transmission however we calibrate for the gap sizes. In Figure S3b we plot the gap size against optical transmission. Notably, maximum transmission is achieved for the smallest gap, which is 200 nm. We use this gap size in our device.

The transmission spectra of the 16 ring resonators at the MUX shown in Figure 1c of the main text are plotted in Figure S3c. 16 distinct resonances are measured at the output (drop) bus, each with minimal overlaps with the others. Every resonance is the output from a distinct PCM device and satisfies the resonance condition $\lambda_m = \frac{2\pi r_m n_{eff}}{m}$, where r_m is the ring radius, m is a positive integer, and n_{eff} is the effective index of the photonic structure. Thus, to adjust the resonance wavelength,

the ring radii are slightly varied resulting in different resonance conditions for each input. Using the above relationship, the ring radii to achieve distinct resonances can be estimated using the expression $\Delta r = \frac{r_1 \Delta\lambda n_{eff}}{\lambda_1 n_g}$. Here r_1 is the radius of the first ring which we used as a reference, λ_1 is its resonance wavelength and n_g is the group index. We choose r_1 = 30 μm, which yields us a sufficient free spectral range, such that the resonances are spectrally separated. For the devices shown in Figure 2e of the main text, the free spectral range is 6.33 nm. All photonic structures are fabricated on a fully etched SiN (Si_3N_4) on SiO_2 wafer, such that the produced strip waveguides are 330 nm in height and 1.2 μm in width. The geometry is larger than the cut-off dimensions required to only support single-mode propagation (TE00), but the couplers are optimized only for (TE00). Thus only single-mode guiding occurs in the waveguides.

PCM device

In Figure S3d, we plot normalized transmission to the output at a fixed wavelength (thus PCM device) for increasing amplitude (energy) of the erase pulses. The 10 nm thin GST patch is 4 μm long and is treated on a hotplate at 250 ^{0}C before optical switching experiments. The measurement thus starts in a fully crystalline state of GST, and for increasing pulses amplitude the device becomes more and more transmissive. This is a result of larger amorphous volumes created in the GST patch, as sketched in the inset cartoons. We note that to achieve a 30% 1.13 dB) change in transmission through amorphization (not shown), a ~12.5 mW optical pulse of 200 ns width and 4 ns edges is sufficient, which corresponds to a switching energy of 2.5 nJ. For the correlation detection experiment, every write signal we use is ~4 mW optical pulse of 200 ns width and 4 ns edges, which corresponds to crystallization energy of 0.8 nJ. For the data shown in Figure S3e, the corresponding accumulative behavior is plotted in Figure S3d. We also point out that our choice of a device with 2 resonators provides the benefits of delineating the phase-transition effects in GST from the resonance conditions of the resonators. In Figure S3f, we plot the transmission spectra of 4 devices before and after switching GST. Notably, the transmission peaks are overlapping each other, which would not have been the case if GST were to be deposited on the resonators (Advanced Optical Materials 5.1 (2017), 1600346).

Optical losses

Considering the device layout sketched in Figure 1b of the main text, the total optical loss in the photonic circuit can be expressed as $\alpha = 10 \log \frac{P_{in}}{P_{out}}$, and estimated by taking to account the different loss mechanisms. This includes propagation losses of SiN waveguide (a_{SiN}), losses from optical absorption by GST (a_{PCM}) it amorphous or crystalline state, coupling (insertion) losses to the MUXs (a_c) and scattering losses at the MUXs (a_s), such that $a = a_{SiN} + a_{PCM} + a_c + a_s$. Alternatively, the loss can be expressed as $P_{in} = P_{out} \times e^{aSiNz} \times e^{aPCMz} \times 2Ne^{ac} \times Ne^{as}$. Here, z is the propagation length of the optical mode and N are the number of devices. The prefactor 2 for coupling losses takes to account the 2 coupling junctions for each resonator (from bus waveguide to the resonator and from the resonator to PCM waveguide). In Figure S3g, we have listed the magnitudes of the different

losses. Note that in our estimations we have excluded losses at the setup (which we measure to be ~-4 dB), and at the grating couplers (which we measure to be ~-6.6 dB/coupler).

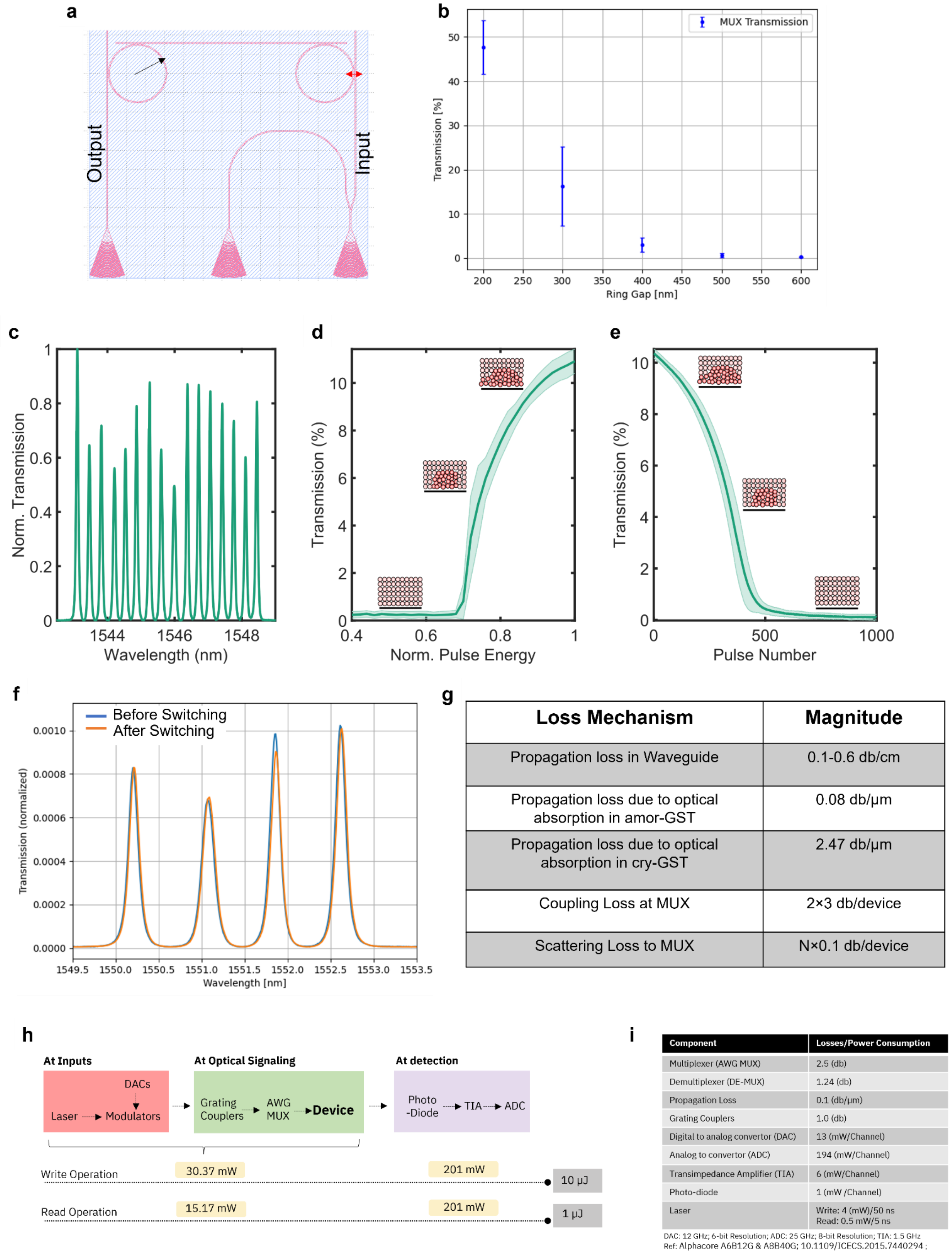

Loss Mechanism	Magnitude
Propagation loss in Waveguide	0.1-0.6 db/cm
Propagation loss due to optical absorption in amor-GST	0.08 db/μm
Propagation loss due to optical absorption in cry-GST	2.47 db/μm
Coupling Loss at MUX	2×3 db/device
Scattering Loss to MUX	N×0.1 db/device

Component	Losses/Power Consumption
Multiplexer (AWG MUX)	2.5 (db)
Demultiplexer (DE-MUX)	1.24 (db)
Propagation Loss	0.1 (db/μm)
Grating Couplers	1.0 (db)
Digital to analog convertor (DAC)	13 (mW/Channel)
Analog to convertor (ADC)	194 (mW/Channel)
Transimpedance Amplifier (TIA)	6 (mW/Channel)
Photo-diode	1 (mW /Channel)
Laser	Write: 4 (mW)/50 ns<br>Read: 0.5 mW/5 ns

DAC: 12 GHz; 6-bit Resolution; ADC: 25 GHz; 8-bit Resolution; TIA: 1.5 GHz
Ref: Alphacore A6B12G & A8B40G; 10.1109/ICECS.2015.7440294 ;

Figure S3: Device characteristics. (a) A schematic of the photonic device. (b) Transmission in the device for differing gap sizes. (c) Transmission spectra of 16 devices. (d) Programming curve of a PCM device. (e)

Accumulation behavior in a PCM device. Inset in (d) and (e) are cartoons of hypothetical phase-configurations. (f) Transmission spectra of 4 devices before and after switching GST. (g) A listing the different loss mechanisms in our photonic circuit. (h) A sketch of the projected energy consumption in different sections of a systems, for Read and Write operations. (i) A listing of different parameters used for the energy.

The energy consumption associated with the photonic computational memory unit is difficult to quantify given that we do not have a complete system yet. However, we can make an estimate based on the existing devices and their energy consumption. The energy consumed per device is approximately 2 nJ for RESET (amorphization), 0.8 nJ for SET (crystallization) switching, and 0.01 nJ for Read Operation. Our previous results (Nature communications, 8, 1-8 (2017)) have improved on the energy consumption to 135 pJ for RESET, 5 pJ for SET switching, and 1 pJ for Read Operation. Considering the latter values and assuming 100 devices (50 are correlated at every timestamp), the total energy consumed for an experiment corresponding to 1 Million Inputs will be 2.50 mJ ($E_{total} = E_{RESET} + E_{SET} + E_{Read} = (100 \times 135$ pJ) + (5×1 pJ $\times 1e6 \times 50$) + (100×1 pJ)). This is comparable to the energy consumed by equivalent electronic phase-change memories. Thus, while benefiting from huge imminent advantages, namely high processing speeds, and parallelization for write and read through wavelength multiplexing, both not possible in the electronic domain, the integrated approach could prove to be equally power efficient. However, note this estimate does not consider the overhead associated with read/write circuitry, data converters, control, and command circuitry as previously mentioned. Supplementary Figures S3(h and i) show the projected power consumption of the different components. The comparison illustrates that energy consumption is dominated by the electronic component (particularly ADCs) in our setup and Photonic Computational Memory minimally contributes to net consumption. In alternate schemes, more efficient ADCs can be considered and the DACs can be simply replaced with a switch, as the mode of operation for correlation detection is binary via Write inputs. Note that the predicted speed based on high-performance photonic integrated photodetectors, modulators, and high efficiency CMOS-based ADC can reach 25 GHz, only to be limited by the crystallization speed of the devices for the Write operation. At first approximation, assuming the crystal growth velocity to 4 nm/ns (Nat Commun 4, 2371 (2013) and each accumulation state requiring a 4 nm region to crystallize, this equates to ~150 MHz operational bandwidth.

S4. Modeling Framework

Finite Element Model Description:

The finite element model simulation framework has been developed using a commercially available FEM software environment (COMSOL®), in combination with a classical nucleation-growth (CNG) cellular automata algorithm. On the FEM model, we initialize the system by the solution of the Helmholtz equation on its port interfaces, to determine the waveguide propagation mode. We use this solution to obtain the spatially and time-resolved distributions of the electromagnetic field (by solution of the wave propagation equation), and the temperature (solving the heat equation via the

input of the optical absorption as the volumetric heat source). The temperature is then used as the driving mechanism inducing the phase transition, calculated via the CNG model, which in turn determines the spatially-resolved material properties of the photonic accumulator GST thin layer.

To drastically reduce the calculation time, the FEM calculation is carried out for the total duration of each pulse, which includes both the pulse time and the cool-down time. The spatially- and time-resolved temperature profile is then extracted to calculate the phase transition taking place during the same time range. This process is repeated iteratively until the completion of the simulated accumulation task. This approximation retains applicability as long as the variation of the crystal fraction (and consequently, of the GST layer optical and thermal properties) is sufficiently low to not induce an appreciable variation of the solutions of the wave and heat equations during the pulse time. Our experimental data shows that ~100 pulses are necessary to achieve the full transmittance drop, thence justifying the use of this approach.

The FEM model geometry replicates a 3D waveguide segment of 8 μm, which includes the waveguide core (SiN), the substrate (SiO_2), and the cladding (air). The photonic accumulator unit cell is placed at the center of the model, on the waveguide top surface, and comprises a GST layer and the SiO_2 capping layer. The GST layer is further fractioned in two distinct volumes, with an (initially) amorphous mark shaped to mimic the experimental results of the SEM characterization, and the remaining fraction fixed to retain the crystalline GST material properties. The amorphous mark assumes an extrusion of the amorphous area identified with the SEM image, across the thickness of the GST layer. The FEM electromagnetic model adopts scattering boundary conditions of the second-order surrounding the model external interfaces, to negate unphysical reflections yet avoiding the use of computationally-intensive PML approaches (https://uk.comsol.com/blogs/). The FEM heat model employs a fixed temperature boundary condition at the external surfaces, which we are allowed to adopt as the distance of those from the heat source is sufficiently large to not determine any significant variation of the heat flux. We note that, since the device is characterized by a planar symmetry, we calculate the model solution on the model of half of the device; on the cut plane, we use the perfect electric conductor approximation to ensure the correct calculation of the waveguide modal profile and the solution for the wave equation. A zero-flux boundary condition is instead applied (on the same cut plane) for the heat model. The material properties utilized in our FEM model are reported in Table S1. In addition to those, thermal contact resistance of 3.5×10^{-8}W/m^{2}K is employed for the GST interfaces with SiN and SiO_2 (Sci. Re p7, 1, 15360, 2017, IEEE Electron Device Lett., 9, 1281–1283, 2011).

The CNG model draws from the classical steady-state description of the nucleation and growth mechanisms. This theory describes the crystallization of an amorphous material starting with the formation of small and unstable clusters, which may either dissolve or grow towards a stable configuration once the temperature-dependent critical size is reached. The driving force of this mechanism is the difference in the Gibbs free energy between the amorphous and the crystalline

phases. The steady-state rates I for nucleation (homogeneous, hom and heterogeneous, het) and growth (gro) are usually expressed as follows:

$$\mathrm{I}_{\mathrm{nuc}}^{\mathrm{ss,hom}} = \mathrm{k}_{\mathrm{nuc}}^{\mathrm{hom}} \exp\left(^{-E_a}/_{k_B T}\right) \exp\left(^{-\Delta \mathrm{G}_{\mathrm{cluster}}}/_{k_B T}\right)$$
$$\mathrm{I}_{\mathrm{nuc}}^{\mathrm{ss,het}} = \mathrm{k}_{\mathrm{nuc}}^{\mathrm{het}} \exp\left(^{-E_a}/_{k_B T}\right) \exp\left(^{-\Delta \mathrm{G}_{\mathrm{cluster}} f(\vartheta)}/_{k_B T}\right)$$
$$\mathrm{I}_{\mathrm{gro}}^{\mathrm{ss}} = \mathrm{k}_{\mathrm{gro}} \exp\left(^{-E_a}/_{k_B T}\right) \phi(T) \left(1 - \exp\left(^{-\Delta \mathrm{G}_{\mathrm{cluster}}}/_{k_B T}\right)\right)$$

where the suffix ss indicates the steady-state value. As visible, each equation is composed by three terms: a kinetic prefactor *k*, an Arrhenius term driven by the activation energy Ea for the diffusion across the phase interface, and a thermodynamic term. In the latter, the critical energy barrier for the cluster formation is $\Delta \mathrm{G}_{\mathrm{cluster}} = \frac{16}{3}\pi \frac{\sigma^3}{\left(\Delta \mathrm{H_f} \frac{T_m - T}{T_m}\right)^2}$ (T_m being GST melting point, and ΔH_f the enthalpy of formation of the crystal phase). The shape factor $f(\vartheta)$, appearing in the heterogeneous process, is usually defined as $f(\vartheta) = \frac{(2-\cos\vartheta)(1-\cos\vartheta)^2}{4}$, with ϑ being the contact angle between the crystallite and the vicinal defect. To improve the adherence of our simulation to the experimental data, we here use also the expression proposed by Peng et al. (J. Appl. Phys., 82, 9, 4183–4191, 1997), $\varphi(\mathrm{T}) = \exp\left(-\frac{0.8}{1-\mathrm{T}/\mathrm{T_m}}\right)$, which is a phenomenological expression which mimics the growth dynamics of thin PCM layers.

In our implementation, we employ a 3D cellular automata framework to manage the temporal evolution and phase-state output of a series of constituent units, defined as monomers, driven by the above-listed equations. The material properties adopted for the CNG model are reported in Table S2; however, more details of the CNG model, and the methods adopted to transfer its output to the FEM model, are discussed in detail in Ref. (E.Gemo,Thesis, 2021). The model here employed does not consider any heterogeneous nucleation contribution, as the literature is incomplete on the quantification of such a phenomenon for a thin GST film sandwiched between a SiN and a SiO_2 layers.

We restrict the calculation to the amorphous mark region, once more for computational requirements reasons. By use of explorative models, we determined the necessary optical power to induce melt-quench on the model (in our case being 4.6 mW). Thus, by setting such a power threshold value as the upper limit in our simulation tasks, we avoid any unexpected melt-quench outside the devised phase-change – "allowed" volume, and therefore any resulting computational artifact, thus validating the applicability of this restriction.

Table S1. Material parameters used for the FEM simulations

material	Refractive index n	Extinction coefficient κ	Density ρ [Kg/m^3]	Specific heat c_P [J/Kg/K]	Thermal conductivity κ_T [W/m/K]
A-GST	3.94† [11]	0.045† [11]	5781 [12], [13]	218 [14]	0.23 [14]
C-GST	6.11† [11]	0.83† [11]	6150 [12]		0.58 [14]
Si_3N_4	2.001† [15]	--	2750 [16]	773† [17]	18.4† [17]
SiO_2	1.445	--	2200 (bulk)	696† [17]	1.37† [17]

The suffix † indicates that the FEM model uses temperature-dependent parameters (room temperature values are reported).

Table S2. Materials and model parameters used for the phase-change simulations

Parameter	value
GST Enthalpy of formation, ΔH_f	1.121×10-9 [J/m3]
Kinetic parameter, hom. nucleation, $\mathrm{k}_{\mathrm{nuc}}^{\mathrm{hom}}$	1.2076×10^{49} [m^3/s]
Kinetic parameter, growth, $\mathrm{k}_{\mathrm{gro}}$	2.0205×10^{8} [m/s]
GST nucleation activation energy, E_a	2.1 [eV]
GST melting temperature, T_m	889 K

We model the accumulation effect induced by delivery of a series of 4.1 mW / 200 ns pulses, consistently with the power used to collect the data shown in Figure S4a, left axis. We here report the plot of the normalized amorphous mark volume (i.e. volume of the amorphous mark divided by its initial volume) as a function of the number of accumulated pulses. As visible, the crystallization process shows the fingerprint trait exhibited by the experimental device, which sees three stages: i) an initial slow-recrystallization stage, ii) a second stage characterized by a steeper crystal fraction decrease, and iii) a third stage where the change of the crystal fraction is again slow.

The 3D plot of the GST phase-state provides a useful lens to this peculiar behavior. Our calculation shows that the initial recrystallization takes place predominantly at the sides of the amorphous mark. This stage is characteristically slow, as the optical-to-thermal energy conversion takes place at the sides of the unit cell, where the optical coupling with the propagating TE_1 mode evanescent field is intrinsically inefficient (see IEEE Trans. Nanotechnol., 10, 4, 900–912, 2011 for more details). However, this holds true until a crystalline bridge is formed at the center of the amorphous mark. At this stage, the unit cell supports a much stronger interaction between GST and the propagating mode, with the temperature peak now closer to the remaining amorphous mark region. During and after this crystalline bridge formation, the rapid recrystallization stage takes place, with relatively faster growth of the crystal phase towards the amorphous region. This process gradually slows as the amorphous mark region is depleted, with the residual A-GST located further

from the optical-to-thermal conversion hot-spot (remaining where the initial crystalline bridge is formed). The maximum temperature data as a function of the pulse number is also reported in Figure S4b, and clearly outlines the rapid shift in temperature which results in the transition among the described crystallization stages.

The simulation data also outlines that, in agreement with our estimation, the recrystallization is strongly growth–driven. In Figure S4a we demonstrate this, by the plot of the normalized crystal fraction (of the investigated amorphous mark region) separated in its nucleation and growth contributions, see the right axis. Here we observe that the growth mechanism is indeed the predominant contribution, with only 7 new nuclei being formed during the simulated accumulation process.

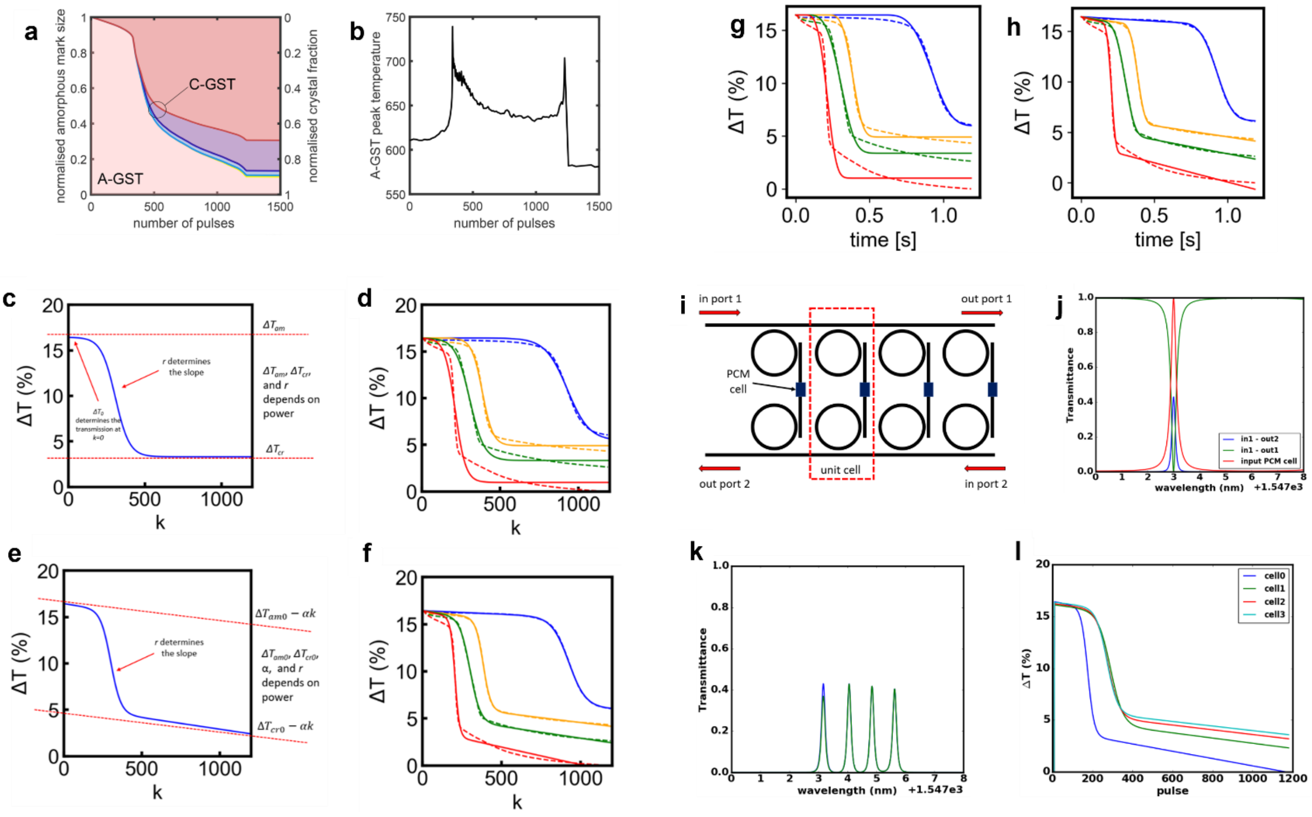

Figure S4. Modelling, Data Fitting and System-Level Simulations. (a) Composition of the analyzed GST volume as a function of the number of pulses. The left axis reports the normalized amorphous mark volume (A-GST, pink area). The right axis reports the corresponding normalized crystal fraction. The other colors appearing on the plot correspond to each one of the stable crystalline nuclei encountered during the accumulation process (for a total of 8 different grains). The simulation data demonstrates how the accumulation process is predominantly growth-dominated. (b) Peak A-GST temperature calculated during the accumulation process, as a function of the number of crystallization pulses. (c) Representation and significance of the fitting parameters in a sample curve produced by the model in Eq. 1. (d) Calculated curves (solid lines) and experimental curves (dashed lines) using the model in Eq. 1 for powers 4.9766 mW (blue), 5.0980 mW (yellow), 5.2194 mW (green), and 5.3408 mW (red). (e) Representation and significance of the fitting parameters in a sample curve produced by the model in Eq. 2 adding the linear component to the logistic function. (f) Calculated curves (solid lines) and experimental curves (dashed lines) using the model in Eq. 2 for powers 4.9766 mW (blue), 5.0980 mW (yellow), 5.2194 mW (green), and 5.3408 mW (red), a noticeable improvement in the fitting of the experimental data is observed when adding the linear component. (g and h) Experimental data for the change in transmittance as a function of time (dashed lines) and fitted data (solid lines) using two different expressions. Traces are for 4.98 mW (blue), 5.10 mW (yellow), 5.22 mW (green), and 5.34 mW crystallization pulses. (i) Diagram of the system being simulated. (j) Frequency response of an isolated unit cell (see i). The transmittance as a function of the wavelength from "in port 1" to "out port 2" (blue line); from "in port 1" to "out port 1" (green line); and from "in port

1" to the input of the PCM cell (red line, note that this will allow us to know the power that the PCM cell is receiving). (k) Frequency response of a system with four unit cells resonant at 1550.17, 1551.06, 1551.85, and 1552.63 nm for the case in which all the phase-change cells are amorphized (blue line), and for the case in which all the cells are amorphized but one that is fully crystalline (green line). (l) Change in transmittance as a function of the pulse number, where the pulse power is 5.3408 mW and for wavelengths corresponding to 1550.17 (blue line), 1551.06 (green line), 1551.85 (red line), and 1552.63 nm (cyan line).

Phenomenological Model:

The time evolution of the change in transmittance ΔT as a function of the pulse number k is going to be given by the following differential equation (Eq. 1):

$$\frac{d\Delta T}{dk} = r(\Delta T - \Delta T_{cr})\left(1 - \frac{\Delta T - \Delta T_{cr}}{\Delta T_{am} - \Delta T_{cr}}\right)$$

In this equation, r is the rate of change, ΔT_{cr} the asymptote for the fully crystalline level, and ΔT_{am} is the asymptote for the fully amorphised level, see Figure S4c. If we integrate this formula we obtain:

$$\Delta T(t) = \frac{1}{e^{-r(k-C)} + \frac{1}{\Delta T_{am} - \Delta T_{cr}}} + \Delta T_{cr}$$

And applying the initial condition $\Delta T(0) = \Delta T_0$, we obtain an expression for the constant:

$$C = \frac{1}{r}\log\left(\frac{1}{\Delta T_0 - \Delta T_{am}} - \frac{1}{\Delta T_{am} - \Delta T_{cr}}\right)$$

Using this expression we can try to fit the experimental data, obtaining the results in Figure S4d. The parameters obtained as a result of this fitting are collected in Table S3.

These results are susceptible to be improved just by adding a linear negative component to Eq. 1. The function describing the data would be then given by (Eq. 2):

$$\Delta T(t) = \frac{1}{e^{-r(k-C)} + \frac{1}{\Delta T_{am0} - \Delta T_{cr0}}} + \Delta T_{cr0} - \alpha k$$

And applying the initial condition $\Delta T(0) = \Delta T_0$, we obtain an expression for the constant:

$$C = \frac{1}{r}\log\left(\frac{1}{\Delta T_0 - \Delta T_{am0}} - \frac{1}{\Delta T_{am0} - \Delta T_{cr0}}\right)$$

In this case, the curve will approach an asymptote with slope given by α and their intercepts are ΔT_{cr0} and ΔT_{am0} for the fully crystalline and amorphized state respectively, see Figure S4e. The fitting using this formula is represented in Figure S4f and the fitting parameters are reported in Table S4.

Table S3. Fitting parameters for curves in Figure S4d from the model using Eq. 1.

	ΔT_{cr}	ΔT_{am}	r
Line 1 (blue)	0.05427162	0.16440019	-1.427641e-2
Line 2 (Orange)	0.04909846	0.16440133	-2.939557e-2
Line 3 (Green)	0.03314425	0.16456956	-2.17343e-2
Line 4 (Red)	0.00970504	0.1646608	-2.993188e-2

Table S4. Fitting parameters for curves in Figure S4f from the model using Eq. 2.

	ΔT_{cr0}	ΔT_{am0}	r	α
Line 1 (blue)	6.90221660e-02	1.64400003e-1	-1.86670428e-02	-7.50113898e-06
Line 2 (Orange)	6.62199605e-02	1.64400012e-1	-4.13942051e-02	-2.05652361e-05
Line 3 (Green)	5.41460542e-02	1.6441011e-1	-3.09090564e-02	-2.50893370e-05
Line 4 (Red)	3.82633208e-02	1.6440007e-01	-6.98856804e-02	-3.73433007e-05

The sigmoidal behavior of crystallization that appears in the experimental data, at first approximation, is similar to the behavior expected in a Avrami framework (JMAK/ Nouv Cim D 20, 1171–1182 (1998)). If we consider the following expression for the crystalline fraction χ(t): $\chi(t) = 1 - \exp\left(-k \cdot (t - t_0)^m\right)$, for $t > t_0$, where the parameters k and m determine the evolution of the crystalline fraction in time (t). We approximate t to be equal to the crystallization pulse number n in our analysis. As well, we introduce an incubation time of t_0. Assuming that the change in transmittance ΔT in the experimental data is directly proportional to the crystallinity χ in the cell, which is a valid assumption according to our results, we could then write the following expression $\Delta T(t) = \Delta T_{cr} - (\Delta T_{cr} + \Delta T_{am0}) \cdot \exp[-k \cdot (t - t_0)^m]$, where ΔT_{am0} is the change in transmittance at $t = 0$, and ΔT_{cr} is the value of the change in transmittance when the crystallization process finishes. See supplementary Figures S4 (g and h). We find the data to not be captured by such a fit. However, we note that the fit improves when a linear negative contribution unique to our photonic devices is added: $\Delta T(t) = \Delta T_{cr} - (\Delta T_{cr} + \Delta T_{am0}) \cdot \exp[-k \cdot (t - t_0)^m] - t\alpha$, for $t > t_0$. This negative term arises because crystallization becomes more and more difficult as the amorphous volume at the input port is approached. This is due to the coupling length required to direct light to GST for optical heating, thus phase transformation, to occur.

Table S5. Fitting parameters for curves in Figure S4h

Curve	ΔT_{cr}	k	t_0	m
Line 1 (blue)	5.956e-2	3.636e1	5.839e-1	3.626
Line 2 (Orange)	4.903e-2	4.083e2	2.138e-1	3.626
Line 3 (Green)	3.375e-2	1.233e2	6.641e-2	3.626
Line 4 (Red)	1.019e-2	5.938e2	5.799e-2	3.626

System Level Simulations:

In this section, we describe how the phenomenological model in the previous section can be integrated within a photonic circuit simulator. Specifically, we refer to the integration in Caphe, a photonic circuit simulator inside the IPKISS 3.3.0 design platform. Using Caphe we are therefore able to define the different components constituents of the circuit (such as waveguides and ring resonators), connect them together and combine their operation with phase-change elements, and extract the frequency and time-domain operation of the whole system.

Definition of waveguides, couplers and ring resonators

The different components are defined by several parameters that describe their behavior. In the case of the waveguides, the specified parameters are the length L of the waveguide, the group index n_g, and the value of $\frac{dn_{\text{eff}}}{d\lambda}$ where n_{eff} are the effective refractive index and λ represents the wavelength (n_g and $\frac{dn_{\text{eff}}}{d\lambda}$ are calculated using FEM obtaining values 2.044 and -3.1x10^5 m^{-1}). Note, that using n_g and $\frac{dn_{\text{eff}}}{d\lambda}$ we can obtain n_{eff} as a function of the wavelength using the expression $n_{\text{eff}}(\lambda) = n_g + \lambda\frac{dn_{\text{eff}}}{d\lambda}$. We also add to the definition of the waveguide the propagation loss reported in Figure S3g of the supplementary information.

In the coupling region of the ring resonators, the relationship between the amplitudes of the modes that are transmitted and coupled to the input mode is done through the parameters t and k respectively. The following relationships are satisfied for the coefficients $|k|^2 + |t|^2 = 1$ and $k = j\sqrt{1-|t|^2}$. In our simulations, we have considered all coupling regions to have the same k. Under this symmetric condition the highest throughput is achieved (assuming low internal losses, which is our case given the low propagation losses of the waveguides). The value for k is extracted using the full width half maximum (FWHM) from the experimental data which was found to be 0.15±0.02 nm. The obtained value for the coupling coefficient is $k = j0.332$, and therefore $t = 0.943$.

The diameters of the ring resonators are around 60 μm, and finely tuned around this value to achieve resonance at the wavelength of interest. Using the length of the ring resonators, the effective refractive index of the waveguides, and assuming we are at 1.55 μm wavelength, we can estimate the resonant order of the resonators which was found to be m=190. When a ring is instantiated in the circuit simulator, we specify the desired resonant wavelength and using the expression $m\lambda = Ln_{\text{eff}}$ we can find the correspondent length/geometry of the ring. We can also

estimate the free spectral range in the ring resonators using the formula $\mathrm{FSR} = \frac{\lambda^2}{n_g L}$. With $\lambda = 1551.5\ \mathrm{nm}$, $n_\mathrm{g} = 2.044$, and $L = \pi \cdot 60\ \mu\mathrm{m}$ we have a calculated FSR=6.248 nm, which matches very well with the experimental value of 6.25 nm.

Frequency and time response of the system:

The simulated system, in this case, allows us to directly compare with the case shown in Figure 2f of the main text. A schematic of such a system is shown in Figure S4i. From the simulation point of view, the system is composed of a unit cell that is successively connected to form the complete system. Each unit cell is composed of two add-drop ring resonators with the geometry appearing in Figure S4j, and both ring resonators are connected by a wavelength containing a phase-change cell. This unit cell will therefore perform an accumulation operation as shown in the previous section but at the wavelength selected by the ring resonators, see Figure S4k. The propagation loss for the fully crystalline phase-change cell is taken from Figure S3c of the supplementary information.

The frequency response of the system when four unit cells resonant at 1550.17, 1551.06, 1551.85, and 1552.63 nm are connected as shown in the main text and we show it here again to properly contextualize the results in this section. We also show the time evolution of the transmittance of each cell when the system is excited with a train of pulses with power 5.3408 mW. More specifically, four simulations are run for an excitation power corresponding to 5.3408 mW and wavelengths corresponding to 1550.17, 1551.06, 1551.85, and 1552.63 nm (resonant wavelength of the four cells) in each one of the four simulations, and the change in transmittance is plotted against the pulse number, see Figure S4l.

In the case of the time-domain simulation the following equation is introduced in the time domain solver of the photonic circuit simulator Caphe, inside IPKISS 3.3.0:

$$\frac{d\Delta T}{dk} = r(\Delta T - \Delta T_{cr})\left(1 - \frac{\Delta T - \Delta T_{cr}}{\Delta T_{am} - \Delta T_{cr}}\right)$$

And adding the linear component $-\alpha k$ to the solution. The parameters ΔT_{cr}, ΔT_{am}, r, and α were obtained by fitting the experimental data, see Table S4, and they are interpolated according to the power arriving at the phase-change cell. Only powers in the interval delimited by the experimental data are used in the simulations. Note also that the equation is defined in terms of k instead of t, which is the variable used for the solver. This is just a scale problem as we can pass from k to t having into account how many seconds a pulse k last. Therefore, we just have to multiply the derivative by a scale factor and run the simulation for the correspondent span.

Section S5. Bag of words approach used in Twitter analysis

In our experiments of correlation detection in live tweets on social media, we used a bag-of-words approach. This approach simplifies the representations used in natural language processing. In this approach, a text (such as a sentence or a tweet) is represented as the bag of its words, disregarding

grammar. Each word is in turn associated with a plurality of other words which all have the same meaning or fit into a context without changing the overall message conveyed in the text. When a tweet arrives at the detector of our computational memory module, it is converted into a list containing the words making the tweet. The list is then compared with the words associated with PCM devices. Each PCM device is associated with not only one keyword but multiple, each given the same significance in our analysis. Every time listed words in the tweet match the catalog of words associated with PCM devices, binary spikes corresponding to write pulses are issued. In Figure S5, we list the words that we associated with individual PCM devices for Figure 4c and Figure 4d in the main text.

a

Device 1 =["VACCINE ";"MEDICATION ";"CURE ";"VACCINATION ";"MEDICINE "];
Device 2 =["DEATH ";"DEATHS ";"DEATHRATE "];
Device 3=["ECONOMY ";"FUNDS ";"CURRENCY ";"FINANCE ";"STOCK MARKET ";"STOCKMARKET ";"DOLLAR ";"FINANCIAL ";"CASH "];
Device 4=["HOPE "; "HOPEFUL ";"HOPING ";"HOPED ";"HOPEFULLY ";"WISH ";"WISHING "]; % Positive Sentiments
Device 5=["VACATION ";"HOLIDAY ";"HOLIDAYS ";"VACATIONS ";"TRAVEL ";"TRAVELLING ";"TOURISM ";"TOURIST ";"TOURISTIC "];
Device 6=["AFRAID ";"FEAR "; "FEARFUL ";"FEARED ";"HORRIFIC ";"HORROR ";"ANXIOUS ";"NERVOUS ";"WORRY ";"WORRISOME ";"WORRIED
Device 7 =["HAPPY ";"HAPPINESS ";"JOY ";"JOYFUL ";"JOYOUS ";"PLEASED ";"CHEERFUL ";"FUN ";"FUNNY "];
Device 8=["QUARANTINE ";"LOCKDOWN ";"SHUTDOWN "];
Device 9=["GOD ";"RELIGION ";"GODLY ";"RELIGIOUS ";"PRAY ";"PRAYER ";"PRAYERS ";"HOLY ";"GODDESS ";"GODDESS ";"WORKSHIP ";"JESUS ";"ALLAH ";"CHURCH ";"MOSQUE "];
Device 10=["TESTING ";"TESTS "];
Device 11=["SAD ";"SORROW ";"SADDENING ";"DEPRESSED ";"DISTRESSED ";"DISTRESS ";"UNHAPPY ";"UNHAPPINESS ";"DEPRESSION ";"DISAPPOINTED ";"STRUGGLE ";"STRUGGLING ";"UPSET ";"DESPAIR ";"SORROWED ";"SADDENED ";"CRYING ";"UPSETTING "];
Device 12=["JOBS "; "JOB "; "UNEMPLOYMENT ";"UNEMPLOYMENTRATE ";"JOBMARKET "];
Device 13=["MASKS ";"SOCIAL DISTANCING "];
Device 14=["SCIENCE ";"RESEARCH "];
Device 15= ["ANGRY ";"FURIOUS ";"OUTRAGED ";"IRKED "; "RAGE "];
Device 16=["RECOVERY ";"RECOVERYRATE ";"IMMUNITY ";"ANTIBODY "];

b

Device 1=["HAPPY ";"HAPPINESS ";"JOY ";"JOYFUL ";"JOYOUS ";"PLEASED ";"CHEERFUL "; "LAUGH "; "FUN "; "FUNNY "; "SMILE ";"HOPE ";"HOPING ";"HOPEFUL "];
Device 2 =["ECONOMY ";"FUNDS ";"CURRENCY ";"STOCK MARKET ";"STOCKMARKET ";"INCOME ";"FINANCE ";"FINANCIAL ";"CASH ";"SALARIES ";"TRADE ";"EXPORT ";"IMPORT "; "UNEMPLOYMENT ";"UNEMPLOYMENTRATE ";"MONEY ";"BUSINESS "];
Device 3 =["HEALTHCARE ";"OBAMACARE ";"MEDICARE ";"VACCINE ";"VACCINATION ";"MEDICATION ";"HEALTH CARE ";"OBAMA CARE ";"MASK";"MASKS ";"SOCIAL DISTANCING ";"MEDICINE ";"HOSPITAL ";"ANTIBODY ";"DRUG ";"DRUGS "];
Device 4 =["COVID ";"COVID-19 ";"CORONAVIRUS ";"PANDEMIC ";"CORONA "];
Device 5 =["ANGRY ";"ANNOY ";"FURIOUS ";"OUTRAGED ";"IRKED "; "RAGE ";"SAD ";"SORROW ";"SADDENING ";"DEPRESSED ";"DISTRESSED ";"DISTRESS ";"UNHAPPY ";"UNHAPPINESS ";"DEPRESSION ";"DISAPPOINTED ";"STRUGGLE ";"STRUGGLING ";"UPSET ";"VIOLENCE ";"FEAR ";"FEARFUL ";"FEARED ";"HORRIFIC ";"HORROR ";"ANXIOUS ";"NERVOUS ";"WORRY ";"WORRISOME ";"WORRIED "];

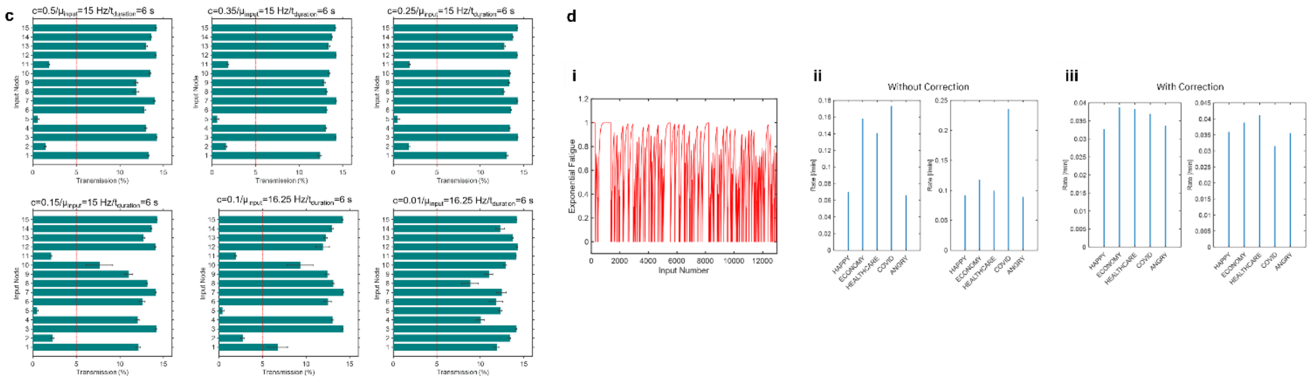

Figure S5: Twitter Analysis. (a) A list of words associated with individual PCM devices for analysis of the subject COVID on Twitter. (b) A list of words associated with individual PCM devices for analysis of the US 2020 elections. (c) Histograms showing the distribution of device transmission values at the end of the experiment for different values of c. (d) (i) A trace showing rate normalization on incoming tweets using exponential averaging. (ii and iii) The rate for incoming keywords on the Republican candidate, without and with (α=0.015) rate correction for two different time-periods of tweets.

The ability to detect temporal correlations depends on the extent of correlation. For example, the task becomes progressively easier with increasing c. Two parameters in our in-memory algorithm importantly govern the efficacy of clustering correlated events from the uncorrelated ones. The first is the variability in the accumulative switching processes across devices. When the switching variability becomes large, events can get incorrectly clustered. For example, for small c the number of correlated events is sparse, thus incomplete switching may -even in a few cycles- lead correlated

devices to not appear correlated. The second is the time-duration of the input streams. For fixed hyperparameters in our algorithm, such as γ and $t_{duration}$, μ_{input}, where $t_{duration}$ and μ_{input} are the period and mean frequency with which input streams arrive, the number of correlated events monotonically reduce as $t_{duration}$ and/or c reduces. This leads to the incomplete crystallization of correlated devices. To illustrate these points, in Figure S5c, using experimental data we simulate different cases for the example of computer networks shown in Section c of the manuscript. The variability in the switching processes is small in our photonic devices and its contribution can be neglected. Thus, the dominating factor affecting the minimal achievable correlation detection is dictated by the time-duration of the input streams. In the top panel of the Figure S5c, the correlator correctly clusters the input streams for correlation coefficients approach c=0.25. For c=0.15 and smaller, however, we note that the increase in the sparsity results in incomplete switching of the correlated devices, i.e. the transmission in correlated devices stays above the threshold. However, when μ_{input} (a proxy for $t_{duration}$) increases, the correlator can detect even smaller coefficients. Because of such dependencies, prior knowledge of the nature of the experiment can be utilized to adjust the algorithm in accordance with the input stream. For example, changes in the hyperparameters and/or use of adaptive threshold for allowing input events that are marginally correlated to get detected.

The α parameter used for normalization of the input rates is fixed in our experiment. However, the parameter can well be adjusted to match the input rates. Supplementary Figures S5c shows the influence of α on the event rates. The data used here the same that is used in Figure 4 of the manuscript.

Section S6. Alternate device architectures

In Figure 1c of the main text, we described the building blocks of our photonic correlation engine, and in the manuscript, we used a chip design with two resonators for DEMUX and DEMUX at the input/drop ports respectively. Here we discuss another design. Here we use ring resonators as MUXs to couple input signals to the PCM devices, but the drop port uses directional couplers of a fixed length. An optical micrograph of such a layout with 4 devices is shown in Figure S6a. In Figure S6b we plot the transmission spectra of such a photonic circuit. We note minimal crosstalk (<-10 dB) between different channels, suggesting our ability to WDM signals.

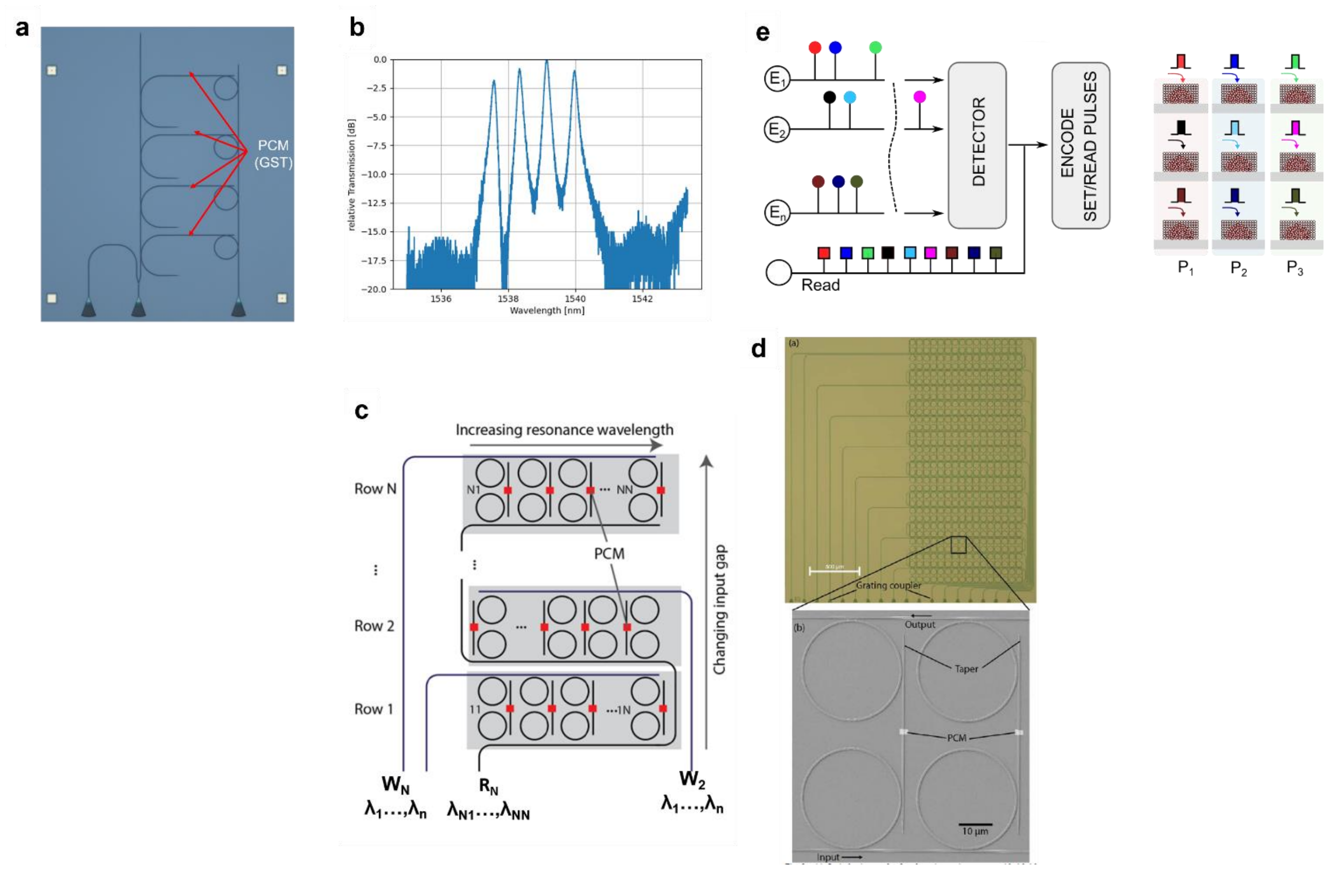

Figure S6: Directional coupler device and photonic array. (a) An optical micrograph of our devices using directional couplers instead of a second resonator at the drop port. (b) Transmission spectra from this device. (c) A conceptual sketch of a photonic circuit layout for incorporating multiple devices. (d) Micrographs of a fabricated chip implementing photonic circuit are shown in (c). (e) Correlation detection in multiple processes. A conceptual sketch of a photonic computational memory module for detecting correlations in multiple processes in parallel using the WDM property.

In the main text, we described proof-of-principle demonstrations with 16 devices. We discuss now a potential architecture that incorporates many more usable devices by still using the same building blocks. A conceptual sketch of this architecture is shown in Figure S6c, which we previously utilized for multi-level memory module applications (IEEE Journal of Selected Topics in Quantum Electronics 26.2 (2019): 1-7). Here we discuss its use of photonic computational memory. The module consists of N rows of the N PCM devices. For consistency with the main text, we consider 16 rows and in each row 16 devices- thus in total 256 devices. Like before, by tuning the radius of the resonators, the wavelength from the input spectrum that is coupled to the PCM device is adjusted. We access the devices for Read operations using the waveguide shown in blue and labeled as R_N. This waveguide carries 16 unique wavelengths. A part of each wavelength signal is dropped by the lower resonators and coupled to the PCM before exiting at the output waveguide, which is shown in blue and is unique for each row. By tuning the coupling gaps between the resonators (both) and waveguides in turn gives control over the amount of light that is coupled. For example, the signal encoded in λ_1 can be coupled equally by the 1st device in every row, such that each drops the amplitude in this signal. This equal splitting can be estimated using the expressions $c_{eff,i} = \frac{1}{N+1-i}$, where N is the number of rows and i the number of the row being addressed. Thus, by using this scheme all devices can be read in parallel. For the write operation, we use the output waveguides,

specific to each row as input. These are shown in blue and labeled as W_N. In this scheme, the output waveguide carries 16 wavelength-multiplexed write signals which are dropped selectively by the top resonators and coupled to the PCM devices. Thus, multiple devices can be also programmed in parallel both within a row and across rows. In Figure S6d, optical and scanning electron micrographs of 256 devices in the above-described format are shown. The overall device has a footprint of 1×2.4 mm^2.

In the main text, we described the possibility to multiplex not only events within a certain process (application) but also processes themselves. This is illustrated in a conceptual sketch shown in Figure S6e. Here the devices are partitioned into different compartments (shown as columns in the figure), where each column is assigned to a certain process. Each process is assigned a set of distinct wavelengths, detectors, and encoders in the digital computational memory module. The WDM property thus allows correlation detection to be performed in parallel across different applications. Experimentally this can be realized using the photonics circuitry shown in Figure S6c. Each row in such a layout can be designated to a unique process and each device in that row to individual events.

Section S7. Additional data on accumulation property

In Figure S7a we plot accumulations as a function of the write pulse amplitude and the number of pulses in a different device to what was shown in the main text. Each data point is averaged over 50 measurements and the data is plotted by taking the amorphous state as a reference (0% transmission). To verify such data sets, in particular the crystallization onset, we performed an additional experiment. In this experiment we amorphized the same device as in (a) to different extents, by modulating the amplitude of the erase pulses (see legend in the plot). Each amorphous state is taken as a reference of 0 % transmission. We then applied to write (crystallization) pulses to the device of increasing amplitude and measured the transmissions change. In Figure S7b we plot such data for 3 distinct initial amorphous volumes (color-coded). We observe that in all three cases the transmission changes from crystallization progressively, before dropping more dramatically beyond some threshold onset. This onset point is independent of the starting state. However, and intuitively the final state achieved is dependent on the starting state, with a larger transmission change achieved in devices programmed originally to larger amorphous volumes.

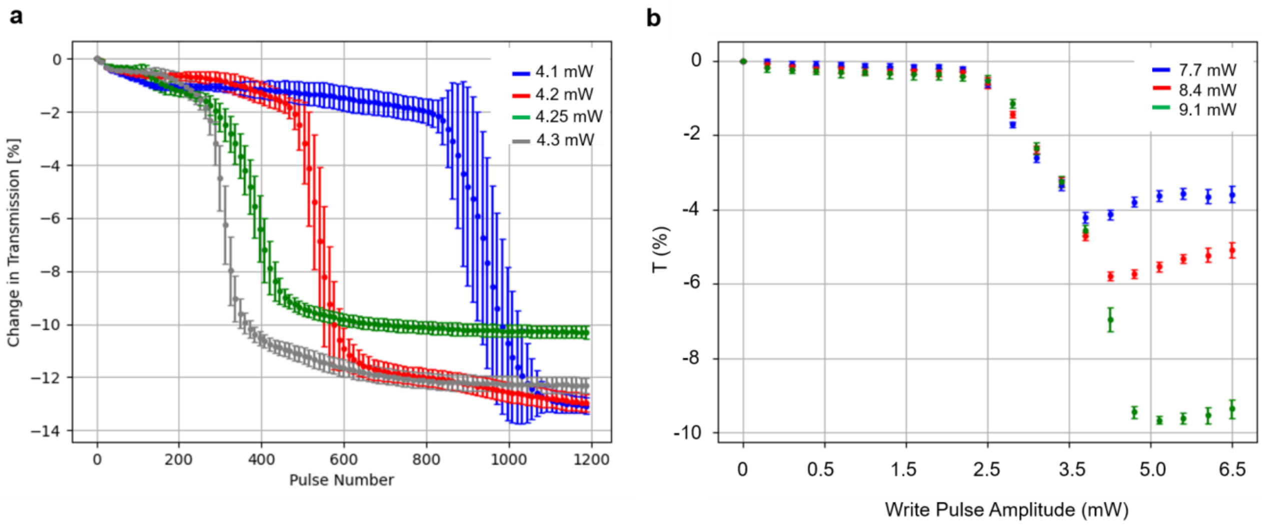

Figure S7: Crystallization behavior of GST. (a) Accumulative behavior in a PCM device. (b) A plot showing progressive crystallization in 3 distinct amorphous phase-configuration of the device.

Movie S1: Simulated crystallization process in photonic GST cell.
